# Transforming traditional physiotherapy hands-on skills teaching into video-based learning

**DOI:** 10.1186/s12909-023-04556-y

**Published:** 2023-09-01

**Authors:** Helena Luginbuehl, Sabine Nabecker, Robert Greif, Stefan Zuber, Irene Koenig, Slavko Rogan

**Affiliations:** 1 School of Health Professions, Division of Physiotherapy, Bern University of Applied Sciences, Murtenstrasse 10, Bern, 3008 Switzerland; 2 Department of Anesthesiology and Pain Management, Sinai Health System, University of Toronto, Toronto, Canada; 3 University of Bern, Bern, Switzerland; 4 School of Medicine, Sigmund Freud University Vienna, Vienna, Austria

**Keywords:** Clinical skills, Physiotherapy education, Student-centered, Skills acquisition, Technology-enhanced

## Abstract

**Background:**

Pandemic-induced restrictions forced curriculum transformation from on-site education to virtual learning options. This report describes this transition, the challenge of creating technology-enhanced learning for hands-on psychomotor skills teaching in physiotherapy, and students’ evaluations of the new technology-enhanced learning approach in Complex Decongestive Physiotherapy.

**Methods:**

On-site theoretical background lectures were replaced with e-learning sessions. Faculty hands-on skills demonstrations for the entire class were replaced with video-recorded demonstrations. Videos included verbal and written instructions and were complemented with checklists guiding the students, training in pairs, through their learning tasks. A cross-sectional observational survey for teaching quality evaluated this new technology-enhanced learning approach and assessed students’ preference for traditional or video-based hands-on skills learning.

**Results:**

Survey return rate was > 50% (46 participating students). Teaching quality was rated between 1.5 ± 0.5 and 1.8 ± 0.4 (Likert scale from − 2 to + 2). Most students (66.7%) preferred the new approach. They appreciated for example that videos were available all the time, enabling self-paced learning, providing an equally good view on skills demonstrations, and the convenience to be able to rewind, re-view, and use speed adjustment options.

**Conclusions:**

Students preferred the new video-based learning of skills for Complex Decongestive Physiotherapy. Because in-class live skills demonstrations were omitted, faculty had more time to provide individual feedback and answer questions. The shift from teacher- to student-centered learning enabled students to control their own learning pace. The innovative program was maintained after pandemic-induced restrictions were lifted. The success of this approach should be tested in other physiotherapy settings and different educational institutions.

**Supplementary Information:**

The online version contains supplementary material available at 10.1186/s12909-023-04556-y.

## Background

Traditionally in physiotherapy education, the process of learning clinical skills is based on didactic classroom lectures conveying theoretical knowledge and subsequent in-person hands-on training sessions with the aim to prepare the students for their clinical rotations [1]. Hands-on clinical skills are physiotherapists’ core competencies, therefore, the teaching and training of required psychomotor skills and their application in practice are of special interest. Students are usually introduced to psychomotor skills, then they practice them in the skills laboratory - supervised by a faculty member who decides upon the sequence of practical competencies that students need to learn. The skills laboratory consists of specifically equipped practice rooms functioning as training facilities, which provide a protected, error-forgiving environment for the practice of clinical skills prior to their application to real patients [1, 2]. In this educational setting, the teaching focus lies predominantly on the psychomotor skills themselves and less on clinical reasoning and the background knowledge of the physiotherapeutic treatment intervention. Physiotherapy students are required to learn to perform psychomotor skills accurately, in a reasonable timeframe, and consistently over time, ideally in a stepwise teaching approach based on an educational framework [3–6]. In physiotherapy education, a faculty member demonstrates a specific physiotherapeutic psychomotor skill to the class of students. All students are required to spread around the therapy table while observing the hands-on demonstration and listening to the faculty member’s explanations. Thereafter, students practice the demonstrated procedure in pairs and receive feedback on their performance from the faculty member. This traditional teaching approach is based on concepts described for teaching and learning clinical skills [3–6]. With this teacher-centered approach [7, 8], the faculty member is at the center of attention, as he or she sets the teaching pace, decides when and how explanations are given and practical skills are demonstrated, as well as when and how feedback is given to students while they are practicing in pairs.

The COVID-19 pandemic led to restrictions to on-site teaching and forced faculties worldwide to incorporate immediate curriculum transformations [9, 10]. Physiotherapy education had to move from on-site education to virtually-provided technology-enhanced learning [11]. A questionnaire of the World Confederation for Physiotherapy on the response to COVID-19 revealed that one of the major challenges at entry level education was hands-on skills teaching, i.e. offering alternative online teaching and learning experiences [12]. At the Bern University of Applied Sciences the development of the technology-enhanced course content had to be completed in a short timeframe, as it was rarely available prior to the COVID-19 pandemic [13].

In this manuscript, we narratively report on the development, curriculum integration, and application of such a new approach for technology-enhanced learning using as an example the Complex Decongestive Physiotherapy lessons in undergraduate physiotherapy education at the Bern University of Applied Sciences, Bern, Switzerland. It incorporates an on-site video-based psychomotor skills learning concept, which is embedded in asynchronous e-learning sessions that teach the theoretical background of applied psychomotor skills [14]. The content of the Complex Decongestive Physiotherapy lessons was the evaluation and management of edema following trauma or orthopedic surgery [15], and chronic lymphedema, e.g., upper extremity lymph edema, following breast cancer surgery and/or radiation [16]. This report summarizes students’ quantitative and qualitative evaluation of their learning with this new technology-enhanced learning concept.

## Methods

### Rationale of the new technology-enhanced skills learning format of Complex Decongestive Physiotherapy

One of many therapy concepts taught in physiotherapy education is Complex Decongestive Physiotherapy [15, 16]: In undergraduate physiotherapy education, this concept contains predominantly practical skills embedded within theoretical teaching sessions. Practical skills include compression bandaging and manual lymphatic drainage.

During on-site teaching restrictions caused by the COVID-19 pandemic in 2020, the classroom lectures of Complex Decongestive Physiotherapy were replaced by asynchronous e-learning sessions. These included short theory inputs (screencasts) which included student activities (e.g., practice questions, processing of patient case examples, quizzes, etc.), formative test questions, and a discussion forum. They were complemented by practical assignments such as self-bandaging or edema assessments. E-learning was provided via the Moodle learning platform (an online learning management system facilitating blended learning and distance education for educational institutions).

Hands-on skills learning-material for these teaching topics was upgraded using some already available short silent videos, to which verbal explanations and written comments were added. Additionally, new videos were produced (Camtasia 2020, TechSmith Cooperation, East Lansing, MI, USA) to facilitate individualized learning on tablets or laptops (https://mediaspace.bfh.ch/media/1.4.+LPT+Kompressionsbandage+Hand_mit+Ton_3_V2/0_a1uahh9h (in German)). After the initial COVID-19 lockdown was lifted, local pandemic precautionary measures to minimize infections allowed the resumption of on-site hands-on practice of essential psychomotor skills by physiotherapy students, in small groups and physically distanced. Practical skills teaching was allowed to be restarted in pairs of students, but live demonstrations by faculty members for the entire class were not yet permitted.

To facilitate such on-site learning, students watched the provided instructional videos in pairs and followed a checklist guiding them through the respective tasks, which they then practiced on each other. A circulating faculty member provided individual feedback and answered any arising questions [13].

This video-based learning approach allowed on-site skills teaching in a classroom format while still keeping physical distance between the pairs of learners to account for infection precaution measures. Regaining on-site education was highly appreciated by students. Benefits reported were: the individualized learning pace, the audio-visual explanations on the instructional videos, that other students were not hindering a direct view of the demonstrated hands-on maneuvers, the possibility to review and adapt the pace of the video, and individual feedback and answers to questions by the faculty member during on-site training [13]. Because of the encouraging feedback on this video-based skills learning concept, and because the asynchronous e-learning sessions on theoretical background rendered formal lectures unnecessary, we further developed and implemented that technology-enhanced format as a regular educational concept for Complex Decongestive Physiotherapy at the Bern University of Applied Sciences after the end of the COVID-19 pandemic-induced restrictions.

### Evaluation of the changed teaching program by survey questionnaires, data collection and analyses

The new technology-enhanced approach was evaluated as a cross-sectional observation using a student survey that comprised two separate questionnaires. Data collection of the survey questionnaires was carried out via the learning platform Moodle and took place during 15 days from June 6th, 2022 (immediately after the last Complex Decongestive Physiotherapy learning session) to June 22nd, 2022. During this period one reminder was sent to students to complete the survey.

Questionnaire A (see Supplementary Material) evaluated the new Complex Decongestive Physiotherapy learning concept (e-learning and video-based hands-on skills learning) using a modified questionnaire developed by Breckwoldt et al. [17]. This questionnaire evaluates teaching quality covering 10 criteria on a Likert scale ranging from − 2 (strongly disagree) to + 2 (strongly agree) [17]. For our evaluation, we used only six items from the original questionnaire [17] including clear structure, climate facilitating learning, clarity of content, individual promotion, effective practicing, and an overall rating. Each item was specified by two examples of teaching behavior (see Supplementary Material).

Questionnaire B (see Supplementary Material) consisted of three self-designed questions on the video-based hands-on skills learning of Complex Decongestive Physiotherapy: (1) A single-choice question looking for preference of the old traditional versus the new video-based skills learning concept. (2) An open question seeking the advantages and disadvantages of video-based versus traditional hands-on skills learning. (3) An open question asking for future improvements of the video-based hands-on psychomotor skills learning.

Quantitative data (participants’ characteristics, questionnaire A and question 1 from questionnaire B) were analyzed by descriptive statistics. Questionnaire A was analyzed using IBM SPSS Statistics for Window Version 28.0 (IBM Corp. Released 2021. Armonk, NY: IBM Corp). Quantitative variables were expressed as means and standard deviations, and percentages. Qualitative results were summarized in Table 1. Since only limited data was gathered, the content of the Table 1 represents all students’ comments summarized and grouped together in themes. A thorough inductive thematic analysis to reach saturation of new data in each theme with the aim to detect patterns that might result in conclusions [18, 19] was impossible to be performed with the data gathered by this short questionnaire.

**Table 1:** Summary of qualitative results of open-answer questions. This represents a summary of all students’ answers grouped together in themes within each of the three questions asked. Due to the limited data available, a thematic analysis to reach saturation was not possible to be performed

Advantages of the video-based skills learning concept	Disadvantages of the video-based skills learning concept	Wishes for improvement of the video-based skills learning concept
**Improvement of learning** - Facilitates self-directed learning - Videos make it easier to learn compared with pictures in a textbook - Possibility to review videos during practical learning - Structured and standardized approach in videos - Videos provide students with an equal, good view of the skills demonstration **Improvement of review** - Videos are available anytime - Easy review and practice, e.g., prior to exams **Improvement of flexibility** - Facilitates remote learning - Facilitates self-paced learning - Possibility to view videos in slow motion for difficult skills	**Missed details** - Live demonstration would enable students to better judge details of a skill, e.g., amount of force needed - Standardized videos might omit discussion of necessary adaptations for individual patients, e.g., obese patients - Focus on the standardized approach in the videos could provoke that students learn the approach by heart rather than understanding the procedure itself - Risks that some errors in the technique might be overlooked and practiced - Some details of procedures might be omitted by students **Missed direct interaction** - Remote learning prevents on-site interaction between students and between students and faculty members **Technical limitations** - Need for headphones to follow videos during practical learning session - Videos can be misleading depending on their quality and angle on the skills demonstration - Videos only provide a 2D perspective, the 3D perspective is missing - Some aspects of a skill might not be as good visible on a video as in real-life demonstrations **Inequality of learning** - Because each student made different progress during learning, it was more complex to follow the skills - Additional information cannot be conveyed to all students at the same time - Risks that not all students perform at the same level at the end of the course - Risk that not all students receive the same input during practical teaching sessions	**Delivery** - Include a live demonstration - Keep the videos for later review - Consider including a session at the start of the practical learning to ensure all students are at the same level - Consider including time at the end of the practice learning sessions to ask questions with the whole group **Content** - Include necessary adaptations for individual patients, e.g., obese patients **Technical** - Allow for a slower pace during the videos, students can adjust the speed themselves if they wish to do so - Improve videos to ensure that all important steps of a skill are clearly visible

### Ethical considerations

The Cantonal Ethics Committee of Bern, Bern, Switzerland reviewed the protocol and exempted the need for approval (BASEC-2022-00701) according to the Federal Act on Research Involving Human Beings as no patients were involved in this research project. This project was conducted according to the current version of the World Medical Association Declaration of Helsinki and the local legally applicable requirements of Switzerland. Informed consent of participating students was obtained with the survey on the Moodle platform.

## Results

Forty-six students, 35 females (76%) and 11 males (24%), aged 23.8 ± 3.4 years enrolled in the Modul BGP2403 Internal Organs 2 at the Bern University of Applied Sciences, BSc Physiotherapy, which includes Complex Decongestive Physiotherapy. Complex Decongestive Physiotherapy comprises six e-learning lessons and 12 on-site video-based hands-on skills lessons.

Twenty-eight students filled in questionnaire A (return rate: 61%), 24 students filled in questionnaire B (return rate 52%).

Table 2 provides the results of the evaluation of questionnaire A. Students rated the teaching quality of this new concept between 1.5 ± 0.5 and 1.8 ± 0.4 on the Likert scale from − 2 to + 2.

**Table 2:** Evaluation of the Complex Decongestive Physiotherapy Questionnaire A (for detailed explanations of the items see Supplementary Material). Ratings of the teaching quality by students in percentage (%), mean and standard deviation

Item	Not sure	Agree	Strongly agree	Mean ± SD
Clear Structure	0.0%	32.1%	67.9%	1.7 ± 0.5
Climate facilitating learning	7.1%	17.9%	75.0%	1.7 ± 0.6
Clarity of content	0.0%	39.3%	60.7%	1.6 ± 0.5
Individual promotion	0.0%	46.4%	53.6%	1.5 ± 0.5
Effective practicing	0.0%	25.0%	75.0%	1.8 ± 0.4
	**Not sure**	**Good**	**Very good**	
Overall rating	0.0%	42.9%	57.1%	1.6 ± 0.5

Seven students (29.2%) preferred the traditional teaching concept while 16 (66.7%) preferred the new video-based learning concept of hands-on psychomotor skills; one student (4.1%) did not answer this question.

Table 1 summarizes the qualitative results of the open-answer questions of questionnaire B. All students’ answers were summarized and grouped together in themes. For example, students appreciated that the videos were available to them at any time, which allowed easy review and repetitive practice, e.g., prior to exams. They appreciated the ability to learn remotely and review the videos during practical learning sessions. Described benefits of the video-based skills learning concept were that it fostered self-paced and self-directed learning and provided all students with the same and equal good view of the skills demonstrations. However, students believe that a live demonstration at the beginning of the skills lesson would enable them to better judge details of a skill procedure, e.g., the amount of force needed for successfully completing a skill.

## Discussion

During the COVID-19 pandemic, new teaching and learning concepts were developed worldwide in a very short time frame involving technology-enhanced learning. This paper presents a newly developed technology-enhanced learning concept for Complex Decongestive Physiotherapy in undergraduate physiotherapy education at the Bern University of Applied Sciences in Switzerland. The COVID-19 pandemic triggered the creation of an ad-hoc new educational concept that was maintained and further developed after the pandemic restrictions were lifted. The new video-based learning approach for Complex Decongestive Physiotherapy was highly favored by students. By leaving out in-class live skills demonstrations, faculty members were able to dedicate more time to offering individual feedback and addressing students’ questions. This transition from teacher- to student-centered learning empowered students to manage their own learning pace.

There is scarce literature evaluating the teaching quality of a technology-enhanced learning concept including video-based hands-on skills learning embedded in e-learning sessions in physiotherapy education. Our results are in line with others, reporting moderate to high levels of physiotherapy undergraduate students’ readiness towards online learning shortly before COVID-19 pandemic restrictions [20]. Other researchers found a similar student satisfaction and even better performance of entry-level undergraduate physiotherapy students regarding online courses compared to face-to-face courses [11]. We discovered a high student satisfaction with the new technology-enhanced psychomotor skills learning concept as more than 60% of the students preferred video-based hands-on skills learning over traditional teaching with faculty skills demonstrations to the whole class.

There is scarce literature on video-based learning of psychomotor skills in physiotherapy. A small pilot study randomized 41 physiotherapy students to face-to-face or video-based learning for a 20-minute session on static and dynamic patellar apprehension tests learning and found similar performance scores in both groups [21]. However, in contrast to our concept, the video-based learning group received only unsupervised training, and therefore no individual feedback regarding their hands-on techniques and lacked the opportunity to ask questions. In this study, student satisfaction was not evaluated. Other researchers [22] compared traditional face-to-face learning with video-based learning of advanced psychomotor skills of cervical spine examination and treatment. Unlike our approach, the videos teaching the respective skill were played twice using the classroom projector system followed by a practice session under faculty supervision and, a subsequent 2-day self-directed, unmonitored practice period. The authors found that online videos were as effective as traditional face-to-face teaching. Another study [23] used video podcasts or vodcasts (video-on-demand-casts) as educational tools for learning clinical physiotherapy musculoskeletal skills, occasionally also used in neurology. Students valued the use of vodcasting for their learning of clinical skills but concluded also that vodcasts should rather be used complementary to faculty demonstrations and not replace them. However, students believed best skills learning would be combining different methods which confirms the movement toward a blended learning approach [24].

In contrast to the “Vodcasting” learning study [23], our teaching program provided students with immediate feedback while learning via the video-based approach by the circulating faculty member (one faculty was in charge of 24 students, which learned in pairs). Another study also evaluated student experience and effectiveness of instructional videos instead of live faculty demonstrations in a second-year osteopathic manipulative medicine course and found that two-thirds of students reported the superiority of videos over faculty demonstrations [25]. This study was more comparable to ours as students followed a worksheet, watched short videos, and then practiced in pairs with individual feedback. Answers to questions were provided by a faculty member, but no demonstrations were provided for the entire class.

Students’ preference of “individual” video-based skills learning compared to faculty member demonstrations for the entire class was confirmed by a clear majority of students in this study [25] as well as in our study. This might be explained by: (1) the highly student-centered approach, (2) the option of individualized learning pace, (3) the multisensory approach, (4) the direct unobstructed and equal view on the clinical skills demonstration, and (5) the individual feedback provided by a faculty member.

### Student-centeredness and individual learning pace

As the new video-based skills learning approach does not provide psychomotor skills demonstrations to the entire class, the faculty member can spend more time for individual student support (giving feedback and answering questions) during the practical training period [21, 26]. Students can control their own learning pace. They can set the speed of the videos, repeat and rewind as necessary and can ask questions and get individual feedback on their practical application as needed [23, 25, 26]. Such a student-centered approach is in line with today’s educational frameworks on adult education as well as a blended learning approach including technology-enhanced learning [21, 23, 27]. Further advantages of the videos are that they can be re-viewed at a later time, e.g., during a clinical internships when students apply such learned skills to real patients [4]. Students from our cohort valued all of those points and also the possibility to view videos in slow motion to master difficult skills.

As reported by others [23], students from our cohort proposed to include live demonstrations of the skills in order not to miss any details or necessary adaptions for certain patients. We initially thought this was not a problem using this new concept as questions that were posed by multiple students in the discussion forum (Moodle) or during the on-site training were later addressed to all students at the beginning of the following skills learning session. However, as the student feedback showed otherwise, this point needs further consideration: E.g., by including very short practical inputs for the entire class resulting from observations of multiple pairs of students during practical training sessions. This would allow providing corrections or mentioning specific approaches and adaptions for certain patients to all students.

Scarce literature addresses the influence of technology-enhanced learning in physiotherapy education on learning pace. In a systematic review [28] the authors conclude that online technologies such as tutorials and website repositories with videos enhanced practical skills performance, but found inconclusive data on the effect on time to perform a given task [28]. We did not evaluate the learning pace of our students. However, our students valued the facilitation of self-paced learning as an advantage of the new learning concept. Interestingly, others perceived this self-paced learning pace as a possible source of inequality of learning (Table 1).

### Multisensory approach and direct view

Skills demonstrations by the faculty member to an entire class often have the effect that some students have different angles of view, and some students might not see the demonstrated psychomotor skill well [25]. This is worsened by increasing class sizes [21]. Instructional videos, like in our concept provided each student with the same clear angle of view and with the same verbal and written comments. All students see the same and get the same information in the same way independent of the learning time of the day. The additional direct feedback by the circulating faculty member during the on-site training completed the multisensory inputs students received through the video-based psychomotor skills learning [23]. Students especially valued the equal good view of the videos provided by our concept (Table 1).

### Individual feedback

An important part of the success of our video-based learning of psychomotor skills was the individual feedback by a faculty member during students’ on-site practice on their task performance [3, 25]. However, in their feedback, as listed in Table 1, students did not comment on the individual feedback other than some mentioned a “risk that not all students receive the same input during practical teaching sessions”. We must consider this not only in video-based but also in traditional skills teaching models. Faculty members give individual feedback to different students, which might not reach all of them. However, traditional teaching included time-intensive demonstrations of the skills resulting in less time for individual feedback to students. Researchers reviewed variables that affect learning and found that observational learning, the external focus of attention, self-controlled practice, and feedback facilitates the learning of motor skills [29]. Our new video-based learning concept matches all those variables. Besides providing information about students’ performance, proper feedback also is a strong motivator with an important positive influence on the learning of those skills [29].

### Strengths and Limitations

There is scarce literature on video-based psychomotor skills learning concepts in physiotherapy education. A systematic review and meta-analysis found a vaguely comparable learning approach which included online video podcasting with no personal faculty member contact for feedback to students’ questions [30]. Strengths of our newly introduced video-based skills learning approach combined with e-learning sessions for the theoretical background are its option for easy curricular integration and its applicability beyond COVID-19 pandemic-induced restrictions. The transferability and easy implementation to further physiotherapeutic fields seem promising as demonstrated in another study introducing a similar concept in a second-year osteopathy medical course, which has many similarities with physiotherapy [25].

Despite the high questionnaire return rate generalizability to other settings might be limited as we report only results from a single center and from a relatively small sample size. Future studies in different physiotherapeutic fields and different settings are therefore needed. Additionally, more, and larger studies need to measure the effect of video-based learning on learning outcomes in comparison to traditional skills teaching and its effect on patient outcomes. Whether the learning videos of the current concept are used in the long run is not yet clarified and might be the subject of future research. A limitation of high-quality videos is the extremely time-consuming production which demands advanced personal competencies and resources. Faculty members need to be trained and supported to develop and apply appropriate technological and pedagogical skills [11].

## Conclusions

We describe an innovative video-based psychomotor skills learning approach in physiotherapy education and its evaluation. Students preferred the video-based approach allowing for self-directed hands-on practice. They especially valued the individual learning pace, the options to adjust the playing speed, the option to repeat and rewind as necessary which enabled individualized learning, and the abundant possibilities to ask questions and to get individual feedback on their practical applications by the faculty member during the on-site skills training session. As skills demonstrations by faculty members to the entire class were no longer necessary, more time for hands-on skills practice with individual feedback and answering students’ questions allowed and fostered a shift from teacher- to student-centered learning. Such a transition is supported by current developments in adult education promoting students’ engagement in their own learning. This promising learning approach and its success in one area of physiotherapy education needs to be validated in broader physiotherapeutic settings and different institutions.

### Electronic supplementary material

Below is the link to the electronic supplementary material.

Supplementary Material 1: Questionnaires A & B.

## Data Availability

The datasets analyzed in the current study are available from the corresponding author on reasonable request.
