# Supplementary material for: Transforming traditional physiotherapy hands-on skills teaching into video-based learning
Source: BMC Med Educ. 2023 Sep 1;23:624. doi: 10.1186/s12909-023-04556-y (PMC10474678; doi:10.1186/s12909-023-04556-y)
Supplement: Supplementary file 1 — Supplementary Material 1: Questionnaires A & B. [file 12909_2023_4556_MOESM1_ESM.pdf]

Supplementary Material

Transforming traditional physiotherapy hands-on skills teaching into video-based learning
H. Luginbuehl, S. Nabecker, R. Greif, S. Zuber, I. Koenig, S. Rogan

Questionnaire A. Modified Questionnaire from Breckwoldt et al. [1]: Original German text/ *(English translation)*

**Sechs allgemeine Feedbackfragen zum Teil Lymphologische Physiotherapie (LPT) im Modul BGP2403 Innere Organe 2** ***(Six general Feedback questions concerning Complex Decongestive Physiotherapy (CDP) of the module BGP2403 Internal Organs 2)***					
-2 "stimme überhaupt nicht zu"; +2: "stimme 100% zu"<br>(*-2 «strongly disagree»; +2 "strongly agree»)*	-2	-1	0	+1	+2
**Klarheit der Struktur *(Clear Structure)***<br>Dieser Oberbegriff beinhaltet z.B.:<br>➢ Während des Moduls war ein "roter Faden" in den LPT-Lernveranstaltungen erkennbar<br>➢ Die Lernschritte bauten thematisch aufeinander auf)<br>*(This item includes e.g.:*<br>➢ *During the module, a central theme was recognizable in the CDP learning events*<br>➢ *The learning steps were thematically built onto each other)*	O	O	O	O	O
**Lernförderliches Klima *(Climate Facilitating Learning)***<br>Dieser Oberbegriff beinhaltet z.B.:<br>➢ Die Studierenden konnten Fragen einbringen<br>➢ Die Studierenden wurden von der Dozentin gleich behandelt<br>*(This item includes e.g.:*<br>➢ *The students could ask questions*<br>➢ *The faculty treated all students equal)*	O	O	O	O	O
**Inhaltliche Klarheit *(Clarity of Content)***<br>Dieser Oberbegriff beinhaltet z.B.:<br>➢ Die Aufgabenstellungen für asynchrone online und praktische vor Ort Lernveranstaltungen waren klar formuliert<br>➢ Die Inhalte der Lernveranstaltungen waren gut verständlich<br>*(This item includes e.g.:*<br>➢ *Assignments for asynchronous online and practical on-site learning sessions were clearly formulated*<br>➢ *The content of the learning sessions was easy to understand)*	O	O	O	O	O
**Individuelles Fördern *(Individual Promotion)***<br>Dieser Oberbegriff beinhaltet z.B.:<br>➢ Alle Studierenden hatten die Möglichkeit sich einzubringen<br>➢ Alle Studierenden erhielten direktes Feedback in den Praxislektionen<br>*(This item includes e.g.:*<br>➢ *All students had the opportunity to get involved*<br>➢ *All students received direct feedback in the practical learning sessions)*	O	O	O	O	O
**Effektives Üben (Effective Practicing)**<br>Dieser Oberbegriff beinhaltet z.B.:<br>➢ Die Übungsaufgaben (z.B. online Übungsfragen, Übungschecklisten) waren für den Lernstand adäquat/ mit dem vorhandenen Wissen bewältigbar<br>➢ Die Dozentin gab gezieltes Feedback und Hilfestellungen<br>*(This item includes e.g.:*<br>➢ *The exercises (e.g., online exercise questions, exercise checklists) were adequate for the learning level/ could be managed with the current knowledge of students*<br>➢ *The faculty member provided targeted feedback and help)*	O	O	O	O	O
**Gesamturteil (Overall rating)**<br>-2 "sehr schlecht"; +2: "sehr gut"; (*-2 «very bad»; +2 "very good»)*	O	O	O	O	O

Questionnaire B. Original German text/ *(English translation)*:

1. Welche Lernform bevorzugen Sie für das Erlernen der Techniken der LPT (Kompressionstherapie und Manuelle Lymphdrainage):

   (Which educational setting do you prefer for learning the Complex Decongestive Physiotherapy skills (manual lymph drainage and compression bandaging:)

   - "Klassischer Unterricht": Vorzeigen der Techniken inkl. mündliche Erklärungen durch die Dozentin und die ganze Gruppe schaut zu. Verbleibende Zeit: Üben in Zweiergruppen mit Feedback durch die Dozentin (als Lernmittel stehen jedoch keine Videos zur Verfügung). Lerntempo ist durch die Dozentin vorgegeben.

     («Traditional teaching»: Demonstration of skills incl. verbal instructions by a faculty member and the entire class watches. Remaining time: Practicing in pairs with feedback from a faculty member (no videos provided as learning aids). Learning pace is driven by the faculty member.)

   - "Video-basiertes Lernen": Techniken inkl. schriftliche und mündliche Erklärungen auf den Videos schauen und Üben in Zweiergruppen mit Feedback durch die Dozentin (kein Vorzeigen in der Grossgruppe resp. höchstens punktuell wenn etwas für die Mehrheit der Klasse unklar ist). Lerntempo erfolgt individuell.

     («Video-based learning»: Watching videos with verbal and written instructions and practicing in pairs receiving feedback from a faculty member (no demonstrations for the entire class, i.e., at most selectively if something is unclear to the majority of the class. Learning pace is individual).

2. Wo sehen Sie Vorteile des "Video-basierten" Lernens im Vergleich zum "klassischen" Physiotherapieunterricht? Wo sehen Sie Nachteile?

   (Where do you see advantages of "video-based" learning compared to "traditional" physiotherapy lessons? Where do you see disadvantages?)

3. Wo sehen Sie Verbesserungsbedarf betreffend des "Video-basierten" Lernens?

   (Where do you see room for improvements regarding "video-based" learning?)
